# ¹H-NMR Metabolic Profiling, Antioxidant Activity, and Docking Study of Common Medicinal Plant-Derived Honey

**DOI:** 10.3390/antiox11101880

**Published:** 2022-09-22

**Authors:** Maha Montaser, Asmaa T. Ali, Ahmed M. Sayed, Usama Ramadan Abdelmohsen, Ehab W. Zidan, Raha Orfali, Mostafa E. Rateb, Mohamed A. Zaki, Hossam M. Hassan, Rabab Mohammed, Mohamed S. Hifnawy

**Affiliations:** 1 Department of Pharmacognosy, Faculty of Pharmacy, Nahda University, Beni-Suef 62513, Egypt; 2 Department of Biochemistry, Faculty of Pharmacy, Nahda University, Beni-Suef 62511, Egypt; 3 Department of Pharmacognosy, Faculty of Pharmacy, Minia University, Minia 61519, Egypt; 4 Department of Pharmacognosy, Faculty of Pharmacy, Deraya University, New Minia City 61111, Egypt; 5 Agricultural Research Centre, Department of Bee Research, Plant Protection Research Institute, Giza 12618, Egypt; 6 Department of Pharmacognosy, College of Pharmacy, King Saud University, Riyadh 11451, Saudi Arabia; 7 School of Computing, Engineering & Physical Sciences, University of the West of Scotland, Paisley PA1 2BE, UK; 8 Department of Pharmacognosy, Faculty of Pharmacy, Beni-Suef University, Beni-Suef 62514, Egypt; 9 Department of Pharmacognosy, Faculty of Pharmacy, Cairo University, Cairo 11787, Egypt

**Keywords:** honey, antioxidant, 5-LOX, metabolomics, NMR, docking

## Abstract

The purpose of this investigation was to determine ¹H-NMR profiling and antioxidant activity of the most common types of honey, namely, citrus honey (HC1) (*Morcott tangerine* L. and *Jaffa orange* L.), marjoram honey (HM1) (*Origanum majorana* L.), and clover honey (HT1) (*Trifolium alexandrinum* L.), compared to their secondary metabolites (HC2, HM2, HT2, respectively). By using a ¹H-NMR-based metabolomic technique, PCA, and PLS-DA multivariate analysis, we found that HC2, HM2, HC1, and HM1 were clustered together. However, HT1 and HT2 were quite far from these and each other. This indicated that HC1, HM1, HC2, and HM2 have similar chemical compositions, while HT1 and HT2 were unique in their chemical profiles. Antioxidation potentials were determined colorimetrically for scavenging activities against DPPH, ABTS, ORAC, 5-LOX, and metal chelating activity in all honey extract samples and their secondary metabolites. Our results revealed that HC2 and HM2 possessed more antioxidant activities than HT2 in vitro. HC2 demonstrated the highest antioxidant effect in all assays, followed by HM2 (DPPH assay: IC_50_ 2.91, 10.7 μg/mL; ABTS assay: 431.2, 210.24 at 50 ug/mL Trolox equivalent; ORAC assay: 259.5, 234.8 at 50 ug/mL Trolox equivalent; 5-LOX screening assay/IC_50_: 2.293, 6.136 ug/mL; and metal chelating activity at 50 ug/mL: 73.34526%, 63.75881% inhibition). We suggest that the presence of some secondary metabolites in HC and HM, such as hesperetin, linalool, and caffeic acid, increased the antioxidant activity in citrus and marjoram compared to clover honey.

## 1. Introduction

Floral honey shows compositional variety, especially in aroma and flavor, because different plants contribute their own bioactive constituents. The fragrant white flowers of *Murcott tangarins*, which are a hybrid of *Citrus reticulate* and *Citrus sinensis* [1], are produced singly or in a cluster of up to six flowers. The flower’s oil consists mainly of linalool, limonene, sabinene, and trans-nerolidol and is used as stomachic carminative, antimicrobial agent, and flavoring agent [2]. The flower of marjoram (*Origanum mjorana*), commonly known as “sweet marjoram”, is a perennial herb native to eastern Mediterranean countries. Marjoram is used worldwide as a spice product. Essential oils from aerial parts of the plants are used in the flavor, perfumery, and pharmaceutical industries. Marjoram is well known for its insecticidal and medicinal value with antioxidant, anticancer, and antimicrobial activities [3,4]. Egyptian clover, *Trifolium alexandrinum* L., is a winter crop widely grown in Egypt, with white or yellow flowers. Its biological activities include antioxidant, anti-inflammatory, anticestodal, and cytotoxic activities, and it is used as a chemoprotective agent against cancers and cardiovascular diseases [4].

Honey is a naturally sweet substance made by honey bees from floral nectar, plant secretions, or plant-sucking bee excretions. After nectar or honeydew collection, transformation by interacting with certain substances in the bee, and maturation, it is then deposited inside the beehive. Honey is produced under different climatic conditions, but the main ingredients in most types of honey are similar.

The process by which nectar saccharides are converted into honey in honeybees comprises regurgitation, evaporation, and enzymatic conversion [5]. One of nature’s most complex foods, honey is the only sweetener that can be ingested without going through a human digestive process [6]. Honey’s energy content is primarily determined by sugars, which account for 95% of its dry weight and are mainly composed of the monosaccharides fructose and glucose [7]. Honey contains roughly 25 oligosaccharides in addition to the two primary sugar components (tri- and tetra-saccharides). The characterization of the honey’s carbohydrate profile was published in different studies [7,8]. Water makes up 12–22% of the composition of honey. Honey’s organoleptic and nutritional qualities are characterized by additional minor components such as organic acids, vitamins, minerals, proteins, amino acids, enzymes, volatiles, and phenolic compounds [9].

Honey’s beneficial health effects, such as its antioxidant, anti-inflammatory, antibacterial, and immune system-stimulating qualities, are due to minor components in addition to its high nutritional value [6,10].

In addition to being a supersaturated solution of glucose and fructose, honey contains 200 other minor metabolites typically present between 0.01 and 10 ppm [11]. Using Nuclear Magnetic Resonance (NMR) spectroscopy to analyze honey has some benefits over other traditional analytical techniques such as GC and GC-MS [12,13]. These include the simultaneous detection of multiple components, the availability of a wealth of information in a single measurement, the high reproducibility and comparability of the data with a high statistical confidence level, and the minimal needs for sample and preprocessing [13,14]. In particular, the metabolomics approach based on NMR spectroscopy, in conjunction with multivariate statistical analysis, is a potent fingerprinting tool that has been effectively utilized for biomarker identification, origin discrimination, and food quality control [15,16,17]. This approach examines metabolite profiles and finds the main discriminating components that differentiate honey varieties. Additionally, numerous studies demonstrated that ^1^H-NMR-based screening techniques are effective tools for the quick examination of honey’s authenticity [13].

The natural antioxidant properties of honey are well recognized; it contains flavonoids, aromatic acids, and polyphenols derived from plants. Other bioactive components such as organic acids, amino acids, vitamins, and proteins are also present [18,19,20]. Esters are essential for honey’s antibacterial and antioxidant activities [21,22]. In addition to phenolics, honey includes enzymes with antibacterial properties, such as glucose oxidase, diastase, invertase, catalase, and peroxidase [18,19,23]. Phenolic acids, flavonoids, vitamins, enzymes, and a trace amount of minerals, mainly copper and iron, are thought to be responsible for honey’s redox properties [24,25]. However, little is understood about the antioxidant properties of honey and the metabolic processes that underlie each component, whether through reducing power or radical scavenging activity, due to their synergistic interactions or the additive combined action of these minor components [10].

The secondary plant metabolites that honey bees acquire with flower nectars are thought to be responsible for honey’s health-promoting qualities. The variety of honey’s secondary metabolites is correlated with its biological activities [26].

Generally, several elements, including botanical, geographic, climatic, and seasonal [27], influence the chemical composition of honey and its quality. Other variables may be external, such as the environment, beekeeper honey treatment practices, storage conditions, and intentional producer adulteration [28]. Several studies have demonstrated that most chronic diseases, including cancer, coronary artery disease, and neurological deterioration, are caused by oxidative damage. Additionally, it has been established that honey’s medicinal efficacy is invariably linked to its antioxidant activity against reactive oxygen species [29]. As a result, current research has concentrated on the composition of the three types of honey and their biological capabilities, including antioxidants, as honey includes a high concentration of free radical scavengers, which support a balance between the levels of antioxidants and free radical production [30]. The high concentration of reducing sugars, more than 65% in honey, such as glucose and fructose, may result in increased reducing antioxidant power in the DPPH method, resulting in a positive error in determining antioxidant activity [29].

Oxidative stress builds up in our bodies as time passes, leading to various illnesses. Oxidative stress may be caused by many metabolic activities within the body and outside stimuli, such as exposure to ultraviolet radiation (UV) and pollutants in the environment [31]. Scientific investigations have shown that free radicals, DNA damage, and cell malignancy are directly linked. Moreover, oxidative stress is involved in the formation of type II diabetes. Because of the significant healthcare costs incurred from these disorders, practical solutions are required to relieve the burdens on people and society. Moreover, the extensive secondary metabolites in honey extracts give them enormous preventive and therapeutic capabilities [32].

More research on phytochemicals has revealed many modern medications, including those now being researched. Natural product-derived bioactive chemicals are more effective therapeutic agents with fewer side effects than synthetics [33]. Polyphenolic natural compounds are a focus of research in both medical supplies and nutrition. In addition to scavenging free radicals, polyphenols may also have potent immunological modulatory and hormone action-inhibitory properties [34]. Polyphenols are also thought to be effective peroxyl radical scavengers, owing to the hydrogen mobility in their molecular structures [21]. Among polyphenols, phenolic acids are perhaps the most abundant in honey. Additionally, they have been observed to affect honey’s flavor and physical appearance, most notably in the color [35].

In this study, we compared the antioxidation potentials of the three most famous regularly used honeys in Egypt (citrus honey, marjoram honey, and trifolium honey—HC1, HM1, and HT1) and their secondary metabolites (HC2, HM2, and HT2). Their radical scavenging potentials were also evaluated using DPPH, APTS, ORAC, and 5-LOX. Additionally, metal chelating activities were also determined, since many free metals have been linked to the production of free radicals [36,37]. The most active compounds suggested in each honey were investigated by the ¹H-NMR fingerprint technique using mathematical models correlating their presence to antioxidant activity. 

In addition, compounds that might contribute to the 5-LOX inhibitory activity of the tested honey samples were predicted depending on a series of in silico and modeling experiments. This work is one of the few studies [38,39] in metabolomics that has attempted to correlate the antioxidant activity of the three most famous types of honey in Egypt to their ¹H-NMR profiles.

## 2. Materials and Methods

### 2.1. Honey Preparation and Collection

#### 2.1.1. Honey Samples

Honey samples were collected in the 2019 season from private apiaries as follows: citrus honey (*Morcott tangerine* L. and *Jaffa orange* L.) from Wadi Almollak, Ismailia Governorate, in April; marjoram honey (*Origanum majorana* L.) from Sawiris Al-Gali Tamiya, Fayoum Governorate, in May; clover honey (*Trifolium alexandrinum* L.) in Mansoura, Dakahlia Governorate, at the end of June. 

#### 2.1.2. Preparation of Reference Slides

A pollen library of all the common plant species found in the honey-producing regions was assembled as a reference library for identifying the pollen extracted from the honey samples. The direct method was used to create reference slides of plant pollen [40]. Fresh plants’ flower buds under study were stripped off their anthers, then washed in an ether-filled watch glass. The ether was decanted, and the pollen was rinsed with fresh ether and left to dry once a ring of pollen had formed at the edge of the ether solution. After being transferred to a microscope slide, the pollen grains were mounted in Kaiser’s glycerin jelly and sealed with paraffin after being warmed to 40 °C.

#### 2.1.3. Qualitative Analysis of Pollen in Honey Samples

The investigation was based on the idea that microscopic elements were concentrated by centrifuging the honey that had been dissolved in water, examining the sediments, and examining them under a microscope. The method for pollen analysis was followed as previously described [41]. Shortly after being dissolved in 20 mL of warm distilled water (about 40 °C), a sub-sample of honey (10 g) was centrifuged twice (at 2000 rpm) for 10 min. After drying with slight heating at 40 °C, the entire sediment was placed on a slide and spread over an area of 20 mm × 20 mm. Glycerin/gelatin was used to mount the sediment, and an alcoholic solution of fuchsin was used to stain it mildly. Slides were examined under a microscope and identified using the reference.

### 2.2. Multivariate and Statistical Analysis

#### 2.2.1. Metabolites Extraction

The secondary metabolite content of honey was extracted using the solid phase extraction (SPE) technique [42]. In brief, 200 g of the available honey samples was thoroughly mixed with 400 mL of deionized H_2_O and 400 mL of MeOH until completely fluid, centrifuged for 10 min at 400 rpm to remove solid particles, and then dried at 40 °C under reduced pressure. Additionally, 100 g of the honey samples was dissolved in 500 mL of acid water (adjusted to pH 2.0). To enable metabolite adsorption, the solution was treated with 100 g of amberlite XAD-LH20 resin (100 m) and gently swirled for 30 min. After stirring, 250 mL of acidic H_2_O and 250 mL of deionized H_2_O were used to wash the resin. To extract the adsorbed metabolites, MeOH (750 mL) was used to wash the resin. As soon as the resin gained its previous white appearance, this step was repeated three to four times. The methanol extract was concentrated at 40 °C in a rotatory evaporator (Buchi, G. Switzerland) before being used for chemical profiling and antioxidant assays [26,43].

#### 2.2.2. H-NMR Analysis

^1^H NMR (400 MHz) spectra were carried out (Bruker, Munich, Germany) using tetramethylsilane TMS as internal standard and dimethyl sulfoxide (DMSO-*d*_6_) as a solvent. Chemical shift values are reported in ppm. Elemental analyses were performed at the Microanalytical Unit, Faculty of Science Ain Shams University.

#### 2.2.3. Multivariate and Statistical Analysis

MetaboAnalyst is a web-based statistical analysis platform that considers ¹H-NMR data. A single zip file comprising the sample name, peak list (ppm), and peak intensities was required for this investigation. The initial step in data normalization was to normalize the raw data using Pareto scaling and the median. After that, multivariate analysis was carried out statistically using unsupervised principal component analysis (PCA) and supervised partial least squares–discriminant analysis (PLS-DA).

### 2.3. Antioxidant Activity

#### 2.3.1. DPPH (Diphenyl-1-picrylhydrazyl) Assay

The 1,1-diphenyl-2-picrylhydrazyl (DPPH) assay, as outlined in [44], was used to measure the honey samples’ efficiency in scavenging free radicals. By dissolving 2 mg in 100 mL of MeOH, the solution (20 mg/L) was created. Then, 0.75 mL of methanolic honey solution was added to 1.5 mL of solution in various concentrations ranging from 20 to 40 mg/mL. The absorbance was measured at 517 nm after 15 min of incubation at 25 °C. The use of ascorbic acid served as a positive control. The ascorbic acid calibration curve (10 and 50 mg/L) was used to calculate the concentration of honey sample needed to scavenge 50% of the ascorbic acid (IC_50_) [45]. The experiment was conducted in triplicate, and the following formula was used to determine the DPPH radical scavenging activity:

(Acontrol − Asample)/(Acontrol × 100) = DPPH radical scavenging activity (percent)

where Asample is the absorbance when a honey extract is present; Acontrol is the absorbance of the control reaction when a honey extract is not present.

#### 2.3.2. ABTS Antioxidant Assay

With a few alterations noted by [46], the method of [47] was applied to assess the free radical scavenging activity. To create the stable ABTS radical cation, the ABTS free radical solution was created (final concentration: 7 mM/L) and incubated for 16 h with potassium persulphate (final concentration: 2.45 mM/L). Five times more ABTS solution was diluted to achieve an absorbance of 2.0–2.4 at 645 nm. In the concentration range of 0 to 0.125 mmol/L, a typical Trolox solution was created. Honey samples were made in water at 1 g/mL concentration. Using a FLUOstar Omega microtiter plate reader (BMG LabTech, Australia) set to 25 °C, 100 mL of ABTS solution was injected into each well of a 96-well flat-bottomed plate. To obtain corrected values, the absorbance of sample and Trolox were measured before and after injection of the ABTS solution. The Trolox-equivalent antioxidant capacity (TEAC) was calculated as mol of Trolox per gram of honey using the following equation, and used to express the antioxidant activity against free radicals.

Scavenging activity (percent) of ABTS = [1 − Ax/A0] × 100

where Ax is the absorbance of the leftover ABTS following the reaction with Trolox and honey solution and A0 is the absorbance obtained using pure water. Trolox percent inhibition was calibrated using a calibration curve. Micromoles of Trolox equivalents per gram of honey (mol TE/g of honey) were used to express the results.

#### 2.3.3. 5-Lipoxygenase Inhibitor Screening Assay

It is known that the oxidation of unsaturated fatty acids containing 1–4 diene groups is catalyzed by lipoxygenase. The appearance of a conjugate diene at 234 nm was used to track the transformation of linoleic acid into 13-hydroperoxy linoleic acid using a UV/visible spectrophotometer. Rutin and nordihydroguaiaretic acid (NDGA), known to inhibit soybean lipoxygenase, were employed as controls. The reaction was started by mixing 2.0 mL of sodium linoleate (100 M) in phosphate buffer with aliquots (50 µL) of daily-prepared lipoxygenase solution at a concentration sufficient to produce an easily quantifiable initial rate of reaction. The enzymatic reactions were carried out in the absence or presence of an inhibitor, and their kinetics were determined. The inhibitors were dissolved in DMSO to the extent that an aliquot (30 µL) produced a final concentration of no more than 100 ppm in each assay. A 30 µL aliquot of the inhibitors yielded a final concentration of no more than 100 ppm in each assay after being thoroughly dissolved in DMSO. The initial reaction rate was calculated using the slope of the straight-line portion of the curve and compared to the control (30 µL of phosphate buffer (pH 9.0) instead of 30 µL of the inhibitor solution) to determine the percentage inhibition of the enzyme activity. The concentration that inhibited 50% of the enzyme (IC_50_) was established by charting the inhibition percentages as a function of the inhibitor concentration [48]. Each inhibitor concentration was tested in triplicate, and the results were averaged (IC_50_ 100 g/mL). Aqueous extracts were not used in this study. A negative lipoxygenase assay result did not always imply that a plant was incapable of acting as an anti-inflammatory agent. Throughout the intricate process of inflammation, the active molecules may have impacts at additional sites [49].

#### 2.3.4. Oxygen Radical Absorbance Capacity (ORAC) Assay

The ORAC test was created as previously reported [50]. AAPH produced free radicals in this experiment, which caused fluorescein to oxidize and lose its fluorescence. Trolox (5 g/mL, final concentration of 20 M) was employed as a reference, and all reagents were made in phosphate buffer (pH 7.0). A final volume of 200 µL test solutions comprising fluorescein (16.7 nM), honey at concentrations ranging from 1 to 10 mg/mL, and AAPH at a concentration of 2.2 mg/mL were placed in each well of the plate reader (Ultimate Concentration). After adding the AAPH, the plate was shaken for 5 s, and fluorescence was observed every 60 s for 110 cycles at wavelengths of 535 and 485 nm for emission and excitation, respectively. ORAC values were calculated using the area under the curve (AUC) method and expressed in mol Trolox equivalent (TE)/g for all fluorescence experiments carried out at 37 °C. A blank containing AAPH, fluorescein, and phosphate buffer was provided (pH 7).

#### 2.3.5. Determination of Metal Chelating Activity

According to previous instructions [51], metal chelating activity was assessed by adding 0.1 mM FeSO4 (0.2 mL) and 0.25 mM ferrozine (0.4 mL) to 0.2 mL of honey extract. The mixture’s absorbance at 562 nm was measured after 10 min of room temperature incubation.

The metal chelating activity is calculated as (Acontrol − Asample)/Acontrol × 100, where Asample is the absorbance in the presence of the extract, and Acontrol is the absorbance of the control reaction (without extract).

#### 2.3.6. Statistical Evaluations of In Vitro Experiments

Each experiment was run three times to ensure accuracy and validity. The mean and standard deviation of three different trials were shown in this example of data presentation. GraphPad 5.0 was used to conduct statistical analysis (GraphPad Software, Inc., La Jolla, CA, USA). Data comparison was performed using the ANOVA, where statistical significance was found to exist when the *p*-value was <0.05.

### 2.4. In Silico and Modeling Investigation

As previously described, binding free energy estimation (∆G binding) and molecular dynamic simulations were performed [52,53]. The Supplementary Materials file has a detailed description of these procedures.

### 2.5. Identification of Isolated Compounds

The methanolic extract was fractionated by column chromatography with silica gel eluted with chloroform/methanol gradient elution 99/1 to 1/1. The highest active fraction (HC2) was subjected to an isolation process; three compounds were isolated and purified by preparative TLC (8:2, *v/v*) chloroform/methanol.

^1^H-NMR spectra of isolated compounds dissolved in DMSO-*d*_6_ were determined with 400 MH_Z_ spectrometers.

## 3. Results

### 3.1. NMR Analysis

**Figure 1:**
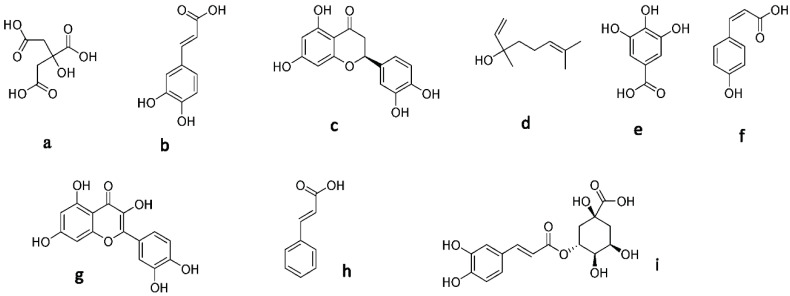
Structure of the secondary metabolites: (**a**) citric acid, (**b**) caffeic acid, (**c**) hesperetin, (**d**) linalool, (**e**) gallic acid, (**f**) *p*-coumaric acid, (**g**) quercetin, (**h**) cinnamic acid, and (**i**) chlorogenic acid.

A range of metabolites have been identified or suggested as being responsible for the antioxidant activity of these types of honey from the literature. ^1^H-NMR analysis of the six honey samples using MestreNova revealed their metabolite profiles. We found that the honey samples showed more antioxidant activity than the honey extract samples. The ¹H-NMR analysis indicated the presence of some minor metabolites in all three types of honey samples (HC2-HM2-HT2), which might be responsible for antioxidant activity (gallic acid, *p*-coumaric acid, quercetin, cinnamic acid, and chlorogenic acid) [54,55,56], and their chemical structures are presented in Figure 1. 

Linalool, hesperetin, and caffeic acid were found only in citrus and marjoram honey, not clover honey. This could be why citrus and marjoram honey samples were more active as antioxidants than trifolium honey [55,57,58,59,60,61].

On the other hand, three compounds were isolated using the column chromatography of HC2 fraction; they were identified comparing their NMR data to the previously reported ones. Compound **1** was characterized as caffeic acid; its ^1^H-NMR spectral data were in good agreement with published data [62,63].

Since ^1^H-NMR spectral data of compound **2** revealed aromatic protons between δ_H_ 6.18 and 7.66 and phenolic OH groups between δ_H_ 9.36 and 12.48, respectively, and agreed with the literature [64,65], it was determined to be quercetin.

Compound **3** was characterized as hesperetin, as its ^1^H-NMR data were in agreement with previous data [66,67].

From these data, we found that citrus honey was more active as an antioxidant then marjoram honey, and the least antioxidant honey was clover or trifolium honey.

### 3.2. Multivariate Data Analysis

**Figure 2:**
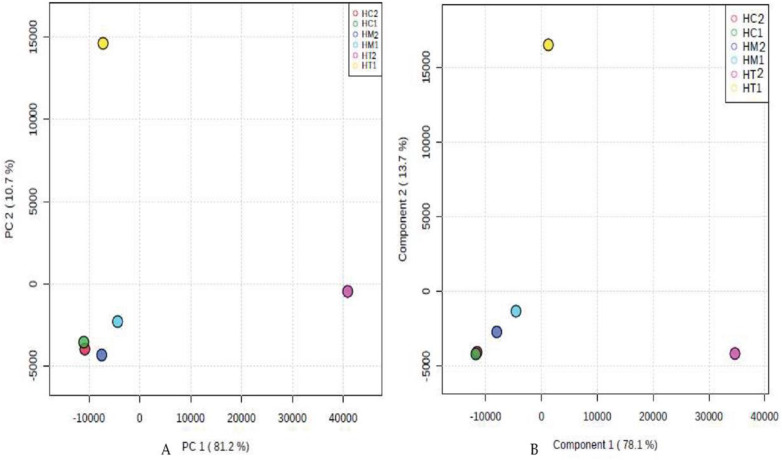
PCA and PLS-DA score plots of the ^1^H-NMR-derived data of the studied honey bee products ((**A**) and (**B**), respectively).

According to the PCA and PLS-DA multivariate analysis (Figure 2), we found that HC2, HM2, HC1, and HM1 were clustered together at PC1 = −10,000, PC2 = −4500 (Figure 2A) and Component 1 = −1000, Component 2 = −4500 (Figure 2B), respectively. However, HT1 and HT2 were plotted far from HC1, HM1, HC2, and HM2. There was a disparity between HC1, HM1, HC2, HM2, and HT1 (10.7% in PCA, 13.7% in PLS-DA) and HT2 (81.2% in PCA, 78.1% in PLS-DA). This finding indicates that HC1, HM1, HC2, and HM2 have similar chemical compositions, while HT1 and HT2 were unique in their chemical profiles.

### 3.3. Antioxidant Capacity

DPPH, ABTS, metal chelating activity, ORAC, and 5-LOX assays were used to evaluate the antioxidant capabilities of honey extracts and their secondary metabolite samples. Trolox, a substitute for vitamin E, resveratrol, and ascorbic acid were employed as standards. For each gram of honey, scavenging capacity was calculated and given in micromoles of reference standard equivalent.

#### 3.3.1. DPPH Radical Scavenging Activity and Metal Chelating Activity

**Figure 3:**
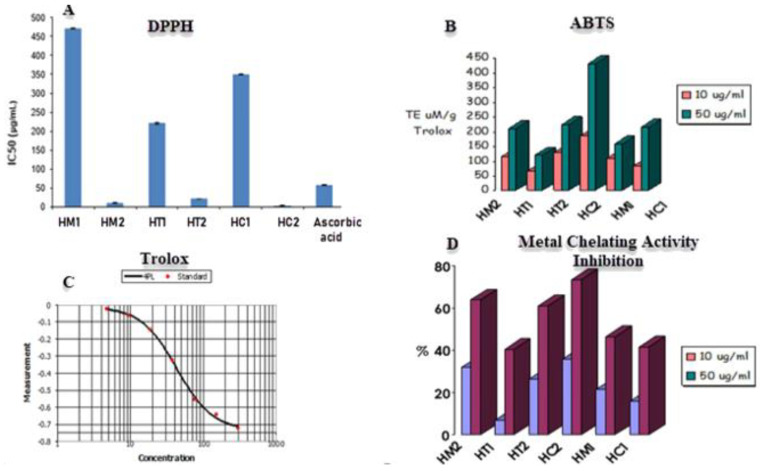
(**A**) DPPH and (**B**) ABTS radical scavenging activities. (**C**) Trolox was used as positive control for SBTS assay. (**D**) Metal chelating activities of different honey extracts. Data are reported as mean ± SE values (*n* = 3).

The DPPH assay was utilized to evaluate the scavenging capacity of honey samples, with ascorbic acid serving as the positive control. The unpaired electron in DPPH reacts with a hydrogen atom provided by honey’s free radical scavenging antioxidant, converting the purple-colored odd electron DPPH to its reduced yellow form. To determine the scavenging ability of honey, the degree of decolorization would be determined using a UV/visible spectrophotometer. The lower the IC_50_ value, the greater the capacity of honey to scavenge radicals, as lowering DPPH requires less radical scavenging capacity from honey. According to Figure 3, the most active scavenging agent is HC2 (citrus honey secondary metabolites) with IC_50_ value of 2.91 μg/mL, followed by HM2 (marjoram honey secondary metabolites), HT2 (trifolium or clover honey secondary metabolites), HT1 (trifolium honey extract), HC1 (citrus honey extract), and HM1 (marjoram honey extract) samples with IC_50_ values of 10.7 μg/mL, 20.5 μg/mL, 220.43 μg/mL, 350.32 μg/mL, and 470.42 μg/mL, respectively.

The antioxidant capacities of the secondary metabolites and honey sample extracts were assessed in relation to various radicals (Figure 3, Table 1). The activity against the ABTS^•+^ radical varied between 66.96 and 185.36 µmol TE μM/10 g Trolox and ranged between 120.48 and 431.2 µmol TE μM/50 g Trolox. For HC2 honey, higher values were reported. Additionally, a metal chelating test using honey extracts was evaluated because excess free irons have been linked to the production and generation of free radicals in biological systems. The six extracts showed substantial chelation activities in concentration-dependent manners, with each sample tested with a concentration of 10 μg/mL and 50 μg/mL (Figure 3). Both concentrations revealed that HC2 and HM2 had the strongest activity with 35.62% and 31.98% inhibition, respectively, while HT1 and HC1 had the least.

#### 3.3.2. ORAC Antioxidant Capacity and Lipoxygenase Inhibition Activity

**Figure 4:**
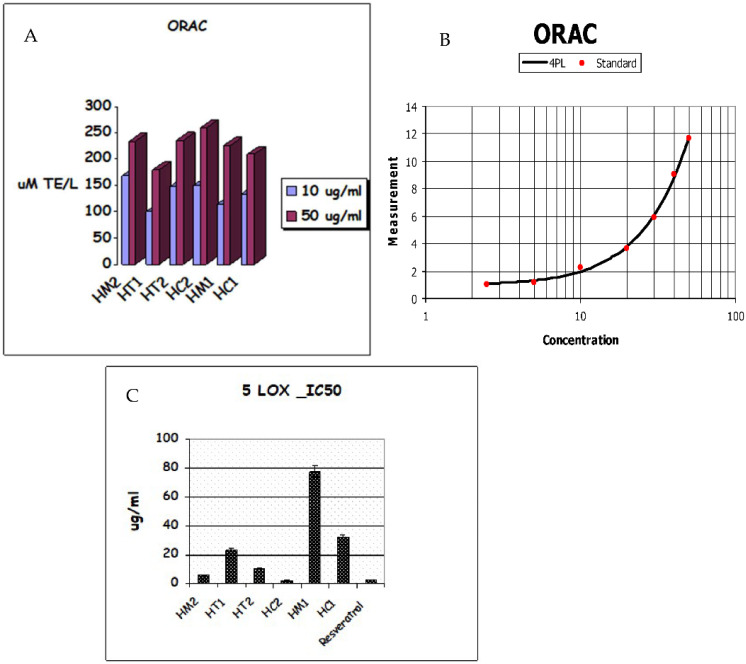
(**A**) ORAC antioxidative activities. (**B**) TE/Trolox standard equivalent. (**C**) Lipoxygenase (5-LOX) inhibition of honey extracts and their metabolites.

The Oxygen Radical Absorbance Capacity (ORAC) experiment revealed that honey samples have the following antioxidant capacities: HC2 > HM2 > HT2 > HM1 > HC1 > HT1. As shown in Table 2, honey extracts showed strong antioxidant activity due to the secondary metabolites in HC2 and HM2, which had respective values of 259.5, 0.448 and 235.8, 1.03 molTE/g, while HC1 and HT1 had ORAC values of 209.7, 0.198 and 180, 0.672 molTE/g, respectively. As shown in Figure 4A,B, HC2 had the greatest ORAC value of all the extracts tested, demonstrating its potential as a free radical scavenger. The investigated honey extracts significantly inhibited 5-lipoxygenase (5-LOX) activity in a similar pattern; among the honey extracts, HC2 (IC_50_ 2.293 g/mL) displayed the highest antioxidant activity (Table 2), while HC1 (IC_50_ 31.87 g/mL) displayed the lowest inhibition of 5-LOX activity (Figure 4C).

### 3.4. Molecular Modeling Study

Several modeling and molecular simulation-based experiments were conducted to determine which compounds might be responsible for the observed inhibitory activity against 5-LOX. First, the structures of all identified compounds (Figure 1) were prepared and docked inside the 5-LOX active site (PDB code: 6N2W). The resulted docking poses for each structure were almost identical; hence, we selected the top-scoring pose for each structure for the subsequent in silico experiment (Table 3). The purpose of the docking step was to putatively generate the static binding mode of each structure inside the enzyme active site. To validate the docking protocol used for the first docking step, the structure of the reported 5-LOX inhibitor, nordihydroguaiaretic acid (NDGA) [68], was re-docked inside the enzyme active site. The produced binding pose was almost identical to that of the co-crystalized one with RMSD of 0.47Å.

Second, generated binding poses from the previous step were used to estimate each structure’s absolute binding free energy (ΔG binding) inside the 5-LOX active site. This step was carried out by conducting a series of molecular dynamics simulations (MDS) according to the free energy perturbation (FEP) protocol [69]. The main purpose of this step was to estimate the relative affinity of each structure towards the enzyme active site. The top three structures (Table 3) with the lowest ΔG binding (<−7 kcal/mol) were then chosen along with NDGA for subsequent 50 ns MDS runs to investigate their stability inside the 5-LOX active site.

Third, caffeic acid, hesperetin, and quercetin showed the highest affinities toward the 5-LOX active site (ΔG binding < −7 kcal/mol); they were then subjected to 50 ns long MDS experiments to explore their dynamic binding stability and mode.

**Figure 5:**
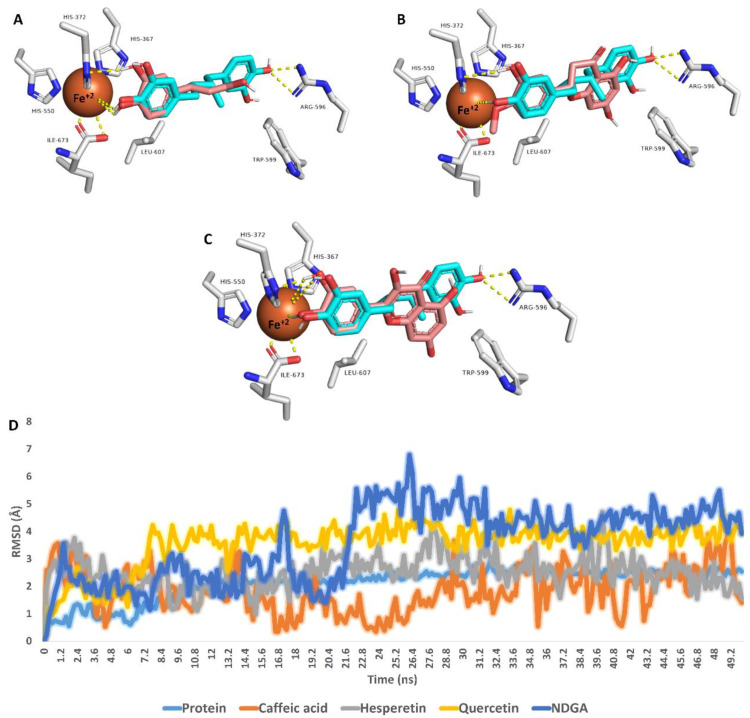
Binding modes of caffeic acid, hesperetin, and quercetin inside the active site of 5-LOX (brick red-colored structures; (**A**–**C**), respectively). Each structure with found to be aligned with NDGA (cyan-colored structure), the reported 5-LOX co-crystallized inhibitor, at their catechol moiety. These binding modes were extracted from the MDS runs as the most populated poses. D is the RMSD of each structure inside the 5-LOX active site over 50 ns long MDS.

The most populated poses were extracted from each MDS run and are depicted in Figure 5. The binding mode of each structure was aligned with that of NDGA to show their degree of similarity, particularly with Fe^+2^ ion. Interestingly, the catechol moiety of the three structures (i.e., caffeic acid, hesperetin, and quercetin) was aligned perfectly with NDGA, establishing H-bonds with HIS-372 and coordinate interactions with Fe^+2^ ion. In addition, only hesperetin and quercetin were able to establish hydrophobic interactions with both TRP-599 and LEU-607.

RMSDs of caffeic acid, hesperetin, quercetin, and NDGA inside the 5-LOX active site ranged from 1.7 Å to 4.1 Å, indicating stability over the simulation. In addition, quercetin showed the lowest fluctuation during the MDS run, while caffeic acid showed the highest. Overall, we could conclude from this modeling and simulation-based experiments that caffeic acid, hesperetin, and quercetin were the potential 5-LOX inhibitors inside the honey samples, particularly HC2, the most potent 5-LOX inhibitor, which was rich in caffeic acid and hesperetin.

## 4. Discussion

From the literature, a range of metabolites have been suggested to be responsible for the antioxidant activity of honey [26]. However, this work is one of the few studies in metabolomics that have attempted to correlate the antioxidant activity of the three most famous types of honey in Egypt—citrus honey (HC1), marjoram honey (HM1), and clover honey (HT1)—compared to their secondary metabolites (HC2, HM2, HT2) by ¹H-NMR profiles, PCA, and PLS-DA multivariate analysis.

Honey’s antioxidant activity is influenced by several parameters, including concentration, temperature, light, substrate type, physical system state, and the existence of micro-components that function as pro-oxidants or synergists [70]. Additionally, it has been proposed that honey’s organic acids, such as gluconic, malic, and citric acids, contribute to antioxidant activity by chelating metals, thus enhancing the activity of flavonoids through synergistic effects [71]. Additionally, the enzymes glucose oxidase and catalase contribute to antioxidant action by their capacity to extract oxygen from the medium [72]. The essential elements of honey responsible for its antioxidant activity include phenolic, flavonoid, and carotenoid concentration, along with ascorbic acid and enzymes related to floral capacity [29,73,74]. 

In this study, we focused on the secondary metabolites that may present in minor concentrations, such as gallic acid, *p*-coumaric acid, quercetin, cinnamic acid, and chlorogenic acid, which might be responsible for antioxidant activity [54,55,56]. To exclude the effect of organic acids and focus on secondary metabolites by using ^1^H-NMR metabolomics, six honey samples were used from crude and prepared honey, and the analysis revealed that their metabolite profiles have more antioxidant activities due to the presence of secondary metabolites compared to the honey extract samples. The ¹H-NMR analysis indicated the presence of these secondary metabolites in all three types of honey samples (HC2, HM2, HT2), whereas linalool, hesperetin, and caffeic acid were found only in citrus and marjoram honey, not clover honey. This could be why citrus and marjoram honey samples were more active as antioxidants than trifolium honey [55,57,58,59,60,61]. Moreover, using PCA and PLS-DA multivariate analysis confirmed our result that HC1, HM1, HC2, and HM2 have similar secondary metabolites, while HT1 and HT2 were unique in their chemical profiles, as we found that HC2, HM2, HC1, and HM1 were clustered together; however, HT1 and HT2 were plotted far from them and each other.

The antioxidant activities of three different types of Egyptian honey (citrus, clover, and marjoram) and their metabolites were evaluated and tested. Clover honey’s antioxidant activity values were generally lower than those of marjoram and citrus honey [29,47,75] based on colorimetrically scavenging activities against DPPH, ABTS, ORAC, 5-LOX, and metal chelating activity in all honey extract samples in concentration-dependent ways. This ideal concentration must be determined to accurately estimate the antioxidant activity of honey types from various floral origins. On the other hand, the relative quantities of minor chemicals, which may be essential to the antioxidant effect, may partially account for the variations in the honey. Numerous authors showed a linear relationship between the amount of all phenolic components and the antioxidant power of plant extracts [47,76,77]. Although their mode of action is unknown, phenolic chemicals and flavonoids are principally responsible for antioxidant activity, with minor chemical compounds [78].

It has been established that 5-LOX contributes to general cellular oxidative stress [79,80]. Accordingly, several previous reports have shown the potential of 5-LOX inhibitors in reducing the 5-LOX-mediated elevated cellular oxidative stress, particularly in inflammatory conditions [81,82], which can lead to cardiovascular, neuronal, and kidney dysfunctions [79,83]. Several theories have been proposed; for example, cinnamon extract activity was indicated as it is linked to free radical sequestration, hydrogen donation, metallic ion chelation, or even has a role as a superoxide or hydroxyl radical substrate. The antioxidant characteristics of these bioactive substances also interfere with propagation processes [84].

Honey’s total phenolic content is essential to correlate to its antioxidant properties. Our current study showed a lower IC_50_ value by HC2 and HM2, suggesting that the sample had more potent antioxidant properties, in agreement with several previous works [21,85]. This showed that flavonoids, along with other honey constituents such as glucose and fructose, could also contribute to the reducing power as one of the primary factors influencing the honey samples’ reduction capacity. More research is needed to determine which phenolic components are responsible for honey’s antioxidant action. Because honey is a complex mix of many different compounds with diverse activity, the involvement of non-phenolic chemicals, which are significant for antioxidant properties, must be examined. Honey also contains amino acids, which have antioxidant properties. Histidine, taurine, glycine, and alanine are a few free amino acids that have antioxidant potential [86]. The relationship between radical scavenging activity and total phenolic content was stronger than between radical scavenging activity and proline content [25]. Honey’s antioxidant action appears complicated because it is connected to various substances, including enzymes, sugars, and plant substrates.

## 5. Conclusions

Our study results reveal that HC2 and HM2 possess the most potential in vitro antioxidant activities. The citrus honey extract (HC2) demonstrated the highest antioxidant activity in all assays (DPPH assay: IC_50_ 2.91 μg/mL; ABTS assay: 431.2 at 50 μg/mL Trolox equivalent; ORAC assay: 259.5 at 50 μg/mL Trolox equivalent; 5-LOX screening assay/IC_50_: 2.293 μg/mL; metal chelating activity at 50 μg/mL: 73.34526% inhibition), followed by HM2 extract (DPPH assay: IC_50_ 10.7 μg/mL; ABTS assay: 210.24 at 50 μg/mL Trolox equivalent; ORAC assay: 234.8 at 50 μg/mL Trolox equivalent; 5-LOX screening assay/IC_50_: 6.136 μg/mL; metal chelating activity at 50 μg/mL: 63.75881% inhibition). 

These results reveal that the secondary metabolites in HC and HM, which were identified as hesperetin, linalool, and caffeic acid, are responsible for increasing the antioxidant activities in citrus and marjoram honey, more than in clover honey. Chromatographing of the HC2 fraction resulted in the isolation and identification of three compounds (caffeic acid, quercetin, and hesperetin) by ^1^H-NMR.

## Figures and Tables

**Table 1:** Free radical scavenging capacities of honey extracts measured with ABTS assay and metal chelating activities at different concentrations on a micro-well plate.

ABTS			Metal Chelating Activity	
TE μM/g Trolox TE μM/g Trolox = 36.56/250 × 1000 = 146.23			% Inhibition	
**Sample**	**10 μg/mL**	**50 μg/mL**	**10 μg/mL**	**50 μg/mL**
HM2	113.88 ± 0.432 ^c^	210.24 ± 1.68 ^c^	31.98978 ± 1.475 ^c^	63.75881 ± 0.7625 ^b^
HT1	66.96± 1.25 ^a^	120.48 ± 1.33 ^b^	6.953549 ± 2.0365 ^a^	40.21628 ± 1.2905 ^a^
HT2	128.96± 0.458 ^c^	224 ± o.655 ^c^	26.50544 ± 1.598 ^c^	60.74911 ± 0.83 ^b^
HC2	185.36± 1.34 ^b^	431.2 ± 2.15 ^a^	35.62371 ± 1.3935 ^c^	73.34526 ± 0.5475 ^c^
HM1	111.0 ± 0.857 ^c^	158.36 ± 0.442 ^b^	21.51157 ± 1.71 ^b^	46.32486 ± 1.1535 ^a^
HC1	83.04 ± 0.612 ^a^	214.76 ± 2.1 ^c^	15.82658 ± 1.835 ^b^	41.68769 ± 1.2575 ^a^
Control	113.88 ± 0.035 ^c^	210.24 ± 0.023 ^c^		

Means with different superscripts (a, b, c) between treatments in the same column are significantly different at *p* < 0.05. Data are represented as mean ± SE values (*n* = 3).

**Table 2:** Free radical scavenging capacities of honey extracts measured with ORAC assay and inhibition (%) of 5-lipoxygenase activity obtained. IC_50_ values represent the mean ± SD of three determinations.

ORAC TE μM/L			5-LOX
Sample	10 μg/mL	50 μg/mL	IC_50_ μg/mL ± SD
HM2	168.3 ± 0.839 ^c^	235.8 ± 1.03 ^c^	6.136 ± 0.4 ^a^
HT1	101 ± 0.606 ^a^	180 ± 0.672 ^a^	23.36 ± 1.4 ^b^
HT2	147.9 ± 0.0776 ^c^	235.4 ± 0.0776 ^c^	10.34 ± 0.6 ^a^
HC2	150.1 ± 0.616 ^c^	259.5 ± 0.448 ^c^	2.293 ± 0.1 ^a^
HM1	115.4 ± 0.175 ^a^	226.8 ± 0.286 ^b^	77.59 ± 4.6 ^c^
HC1	134.7 ± 0.69 ^b^	209.7 ± 0.198 ^b^	31.87 ± 1.9 ^b^
NDGA			2.696 ± 0.2 ^a^

Means with different superscripts (a, b, c) between treatments in the same column are significantly different at *p* < 0.05. Data are represented as mean ± SD values (*n* = 3).

**Table 3:** Docking and ΔG binding scores of the identified compounds inside the active site of 5-LOX, along with their H-bonding and hydrophobic interactions.

Structure	Docking Score (kcal/mol)	ΔG Binding (kcal/mol)	Average RMSD (Å)	H-Bonding	Hydrophobic Interaction
Caffeic acid	−7.1	−7.5	1.7	HIS-372	LEU-607
Hesperetin	−7.5	−7.9	2.8	HIS-372	TRP-599, LEU-607
Quercetin	−7.6	−8.4	4.0	HIS-372	TRP-599, LEU-607
Chlorogenic acid	−6.1	−5.3	>5	ARG-596	TRP-599
Cinnamic acid	−6.9	−4.7	>5	-	TRP-599, LEU-607
*p*-coumaric acid	−6.2	−4.1	>5	ARG-596	TRP-599
Citric acid	−5.3	−3.6	>5	ARG-596, TRP-599	-
Gallic acid	−4.7	−2.3	>5	TRP-599	-
Linalool	−3.5	−1.1	>5	-	TRP-599
NDGA *	−7.9	−8.6	4.7	HIS-372, ARG-596	TRP-599, LEU-607

* NDGA is the previously reported 5-LOX co-crystallized inhibitor [68].

## Data Availability

Data is contained within the article and Supplementary Materials.

## Supplementary Materials

The following supporting information can be downloaded at: https://www.mdpi.com/article/10.3390/antiox11101880/s1, Figure S1. ¹HNMR chart of Citrus honey extract and its 2nd metabolites (H1 and Hc); Figure S2. ¹HNMR chart of Marjoram honey extract and its 2nd metabolites (H2 and HM); Figure S3. ¹HNMR chart of Clover honey extract and its 2nd metabolites (H3 and HT); Figure S4. ¹HNMR chart of compound no 2: Qurcetin; Figure S5. ¹HNMR chart of compound no 3: Hesperetin; Figure S6. ¹HNMR chart of compound no 1: Caffeic acid. Refs. [87,88,89,90,91] in Supplementary Materials.

## References

[1] Feger W., Brandauer H., Ziegler H. (2003). Analytical Investigation of Murcott (Honey) Tangerine Peel Oil. J. Essent. Oil Res..

[2] Tan F.C., Swain S.M. (2007). Functional Characterization of AP3, SOC1 and WUS Homologues from Citrus (*Citrus sinensis*). Physiol. Plant..

[3] Gharib F.A., Badr S.E.A., Al-Ghazali B.A.S., Zahran M.K. (2013). Chemical Composition, Antioxidant and Antibacterial Activities of Lavender and Marjoram Essential Oils. Egypt. J. Chem..

[4] Abdalla M.M.F., Abd El-Naby Zeinab M. (2012). Inbreeding and Fertility in Egyptian Clover, Trifolium Alexandrinum. J. Pharmacogn. Phyther..

[5] Jamróz M.K., Paradowska K., Zawada K., Makarova K., Kaźmierski S., Wawer I. (2014). ^1^H and ^13^C NMR-Based Sugar Profiling with Chemometric Analysis and Antioxidant Activity of Herbhoneys and Honeys. J. Sci. Food Agric..

[6] Kazalaki A., Misiak M., Spyros A., Dais P. (2015). Identification and Quantitative Determination of Carbohydrate Molecules in Greek Honey by Employing ^13^C NMR Spectroscopy. Anal. Methods.

[7] Consonni R., Cagliani L.R., Cogliati C. (2012). NMR Characterization of Saccharides in Italian Honeys of Different Floral Sources. J. Agric. Food Chem..

[8] Pascual-Maté A., Osés S.M., Marcazzan G.L., Gardini S., Muiño M.A.F., Sancho M.T. (2018). Sugar Composition and Sugar-Related Parameters of Honeys from the Northern Iberian Plateau. J. Food Compos. Anal..

[9] Bogdanov S., Jurendic T., Sieber R., Gallmann P. (2008). Honey for Nutrition and Health: A Review. J. Am. Coll. Nutr..

[10] Gheldof N., Wang X.-H.H., Engeseth N.J. (2002). Identification and Quantification of Antioxidant Components of Honeys from Various Floral Sources. J. Agric. Food Chem..

[11] Ciulu M., Spano N., Pilo M.I., Sanna G. (2016). Recent Advances in the Analysis of Phenolic Compounds in Unifloral Honeys. Molecules.

[12] Karabagias I.K., Vlasiou M., Kontakos S., Drouza C., Kontominas M.G., Keramidas A.D. (2018). Geographical Discrimination of Pine and Fir Honeys Using Multivariate Analyses of Major and Minor Honey Components Identified by ^1^H NMR and HPLC along with Physicochemical Data. Eur. Food Res. Technol..

[13] Spiteri M., Jamin E., Thomas F., Rebours A., Lees M., Rogers K.M., Rutledge D.N. (2015). Fast and Global Authenticity Screening of Honey Using ^1^H-NMR Profiling. Food Chem..

[14] Schievano E., Stocchero M., Morelato E., Facchin C., Mammi S. (2012). An NMR-Based Metabolomic Approach to Identify the Botanical Origin of Honey. Metabolomics.

[15] Consonni R., Cagliani L.R. (2008). Geographical Characterization of Polyfloral and Acacia Honeys by Nuclear Magnetic Resonance and Chemometrics. J. Agric. Food Chem..

[16] Beretta G., Caneva E., Regazzoni L., Bakhtyari N.G., Facino R.M. (2008). A Solid-Phase Extraction Procedure Coupled to ^1^H NMR, with Chemometric Analysis, to Seek Reliable Markers of the Botanical Origin of Honey. Anal. Chim. Acta.

[17] Girelli C.R., Del Coco L., Fanizzi F.P. (2016). ^1^H NMR Spectroscopy and Multivariate Analysis as Possible Tool to Assess Cultivars, from Specific Geographical Areas, in EVOOs. Eur. J. Lipid Sci. Technol..

[18] Miguel M.G., Antunes M.D., Faleiro M.L. (2017). Honey as a Complementary Medicine. Integr. Med. Insights.

[19] Combarros-Fuertes P., Fresno J.M., Estevinho M.M., Sousa-Pimenta M., Tornadijo M.E., Estevinho L.M. (2020). Honey: Another Alternative in the Fight against Antibiotic-Resistant Bacteria?. Antibiotics.

[20] Kuś P.M. (2020). Honey as Source of Nitrogen Compounds: Aromatic Amino Acids, Free Nucleosides and Their Derivatives. Molecules.

[21] Al-Mamary M., Al-Meeri A., Al-Habori M. (2002). Antioxidant Activities and Total Phenolics of Different Types of Honey. Nutr. Res..

[22] Sateriale D., Facchiano S., Colicchio R., Pagliuca C., Varricchio E., Paolucci M., Volpe M.G., Salvatore P., Pagliarulo C. (2020). In Vitro Synergy of Polyphenolic Extracts from Honey, Myrtle and Pomegranate against Oral Pathogens, *S. mutans* and *R. dentocariosa*. Front. Microbiol..

[23] Ahmed S., Sulaiman S.A., Baig A.A., Ibrahim M., Liaqat S., Fatima S., Jabeen S., Shamim N., Othman N.H. (2018). Honey as a Potential Natural Antioxidant Medicine: An Insight into Its Molecular Mechanisms of Action. Oxid. Med. Cell. Longev..

[24] Erlund I. (2004). Review of the Flavonoids Quercetin, Hesperetin, and Naringenin. Dietary Sources, Bioactivities, Bioavailability, and Epidemiology. Nutr. Res..

[25] Meda A., Lamien C.E., Romito M., Millogo J., Nacoulma O.G. (2005). Determination of the Total Phenolic, Flavonoid and Proline Contents in Burkina Fasan Honey, as Well as Their Radical Scavenging Activity. Food Chem..

[26] Suarez A.F.L., Tirador A.D.G., Villorente Z.M., Bagarinao C.F., Sollesta J.V.N., Dumancas G.G., Sun Z., Zhan Z.Q., Saludes J.P., Dalisay D.S. (2021). The Isorhamnetin-Containing Fraction of Philippine Honey Produced by the Stingless Bee *Tetragonula biroi* Is an Antibiotic against Multidrug-Resistant *Staphylococcus aureus*. Molecules.

[27] Nicolson S.W., Nepi M., Pacini E. (2007). Nectaries and Nectar.

[28] Soria A.C., González M., De Lorenzo C., Martınez-Castro I., Sanz J. (2004). Characterization of Artisanal Honeys from Madrid (Central Spain) on the Basis of Their Melissopalynological, Physicochemical and Volatile Composition Data. Food Chem..

[29] Ferreira I.C.F.R., Aires E., Barreira J.C.M., Estevinho L.M. (2009). Antioxidant Activity of Portuguese Honey Samples: Different Contributions of the Entire Honey and Phenolic Extract. Food Chem..

[30] Kishore R.K., Halim A.S., Syazana M.S.N., Sirajudeen K.N.S. (2011). Tualang Honey Has Higher Phenolic Content and Greater Radical Scavenging Activity Compared with Other Honey Sources. Nutr. Res..

[31] Murapa P., Dai J., Chung M., Mumper R.J., D’Orazio J. (2012). Anthocyanin-rich Fractions of Blackberry Extracts Reduce UV-induced Free Radicals and Oxidative Damage in Keratinocytes. Phyther. Res..

[32] Ibrahim M.A., Koorbanally N.A., Kiplimo J.J., Islam M.S. (2012). Anti-Oxidative Activities of the Various Extracts of Stem Bark, Root and Leaves of *Ziziphus Mucronata* (Rhamnaceae) in Vitro. J. Med. Plants Res..

[33] Facchini P.J., De Luca V. (2008). Opium Poppy and Madagascar Periwinkle: Model Non-model Systems to Investigate Alkaloid Biosynthesis in Plants. Plant J..

[34] Havsteen B.H. (2002). The Biochemistry and Medical Significance of the Flavonoids. Pharmacol. Ther..

[35] Steeg E., Montag A. (1988). Minor Ingredients of Honey with Flavor Relevancy. 2. Sensorially Active Decomposition Products of Carboxylic-Acids and Glycosidically Bonded Aromates. Dtsch. Leb..

[36] Martemucci G., Costagliola C., Mariano M., D’andrea L., Napolitano P., D’Alessandro A.G. (2022). Free Radical Properties, Source and Targets, Antioxidant Consumption and Health. Oxygen.

[37] Phaniendra A., Jestadi D.B., Periyasamy L. (2015). Free Radicals: Properties, Sources, Targets, and Their Implication in Various Diseases. Indian J. Clin. Biochem..

[38] Alhadrami H.A., Sayed A.M., El-Gendy A.O., Shamikh Y.I., Gaber Y., Bakeer W., Sheirf N.H., Attia E.Z., Shaban G.M., Khalifa B.A. (2021). A Metabolomic Approach to Target Antimalarial Metabolites in the Artemisia Annua Fungal Endophytes. Sci. Rep..

[39] More G.K., Meddows-Taylor S., Prinsloo G. (2021). Metabolomic Profiling of Antioxidant Compounds in Five Vachellia Species. Molecules.

[40] Sawyer R., Pickard R.S. (1981). Pollen Identification for Beekeepers.

[41] Louveaux J., Maurizio A., Vorwohl G. (1978). Methods of Melissopalynology. Bee World.

[42] Ferreres F., Tomáas-Barberáan F.A., Gil M.I., Tomáas-Lorente F. (1991). An HPLc Technique for Flavonoid Analysis in Honey. J. Sci. Food Agric..

[43] Ferreres F., Andrade P., Tomfis-Barberfin F.A. (1994). Flavonoids from Portuguese Heather Honey.

[44] Isla M.I., Craig A., Ordoñez R., Zampini C., Sayago J., Bedascarrasbure E., Alvarez A., Salomón V., Maldonado L. (2011). Physico Chemical and Bioactive Properties of Honeys from Northwestern Argentina. LWT Food Sci. Technol..

[45] Barbosa-Pereira L., Angulo I., Paseiro-Losada P., Cruz J.M. (2013). Phenolic Profile and Antioxidant Properties of a Crude Extract Obtained from a Brewery Waste Stream. Food Res. Int..

[46] Brangoulo H.L., Molan P.C. (2011). Assay of the Antioxidant Capacity of Foods Using an Iron (II)-Catalysed Lipid Peroxidation Model for Greater Nutritional Relevance. Food Chem..

[47] Baltrušaitytė V., Venskutonis P.R., Čeksterytė V. (2007). Radical Scavenging Activity of Different Floral Origin Honey and Beebread Phenolic Extracts. Food Chem..

[48] Njenga E.W., Viljoen A.M. (2006). In Vitro 5-Lipoxygenase Inhibition and Anti-Oxidant Activity of *Eriocephalus* L. (Asteraceae) Species. S. Afr. J. Bot..

[49] Akula U.S., Odhav B. (2013). In Vitro 5-Lipoxygenase Inhibition of Polyphenolic Antioxidants from Undomesticated Plants of South Africa. J. Med. Plants Res..

[50] Gillespie K.M., Chae J.M., Ainsworth E.A. (2007). Rapid Measurement of Total Antioxidant Capacity in Plants. Nat. Protoc..

[51] Chew Y.-L., Goh J.-K., Lim Y.-Y. (2009). Assessment of in Vitro Antioxidant Capacity and Polyphenolic Composition of Selected Medicinal Herbs from Leguminosae Family in Peninsular Malaysia. Food Chem..

[52] Alhadrami H.A., Alkhatabi H., Abduljabbar F.H., Abdelmohsen U.R., Sayed A.M. (2021). Anticancer Potential of Green Synthesized Silver Nanoparticles of the Soft Coral Cladiella Pachyclados Supported by Network Pharmacology and In Silico Analyses. Pharmaceutics.

[53] Alhadrami H.A., Burgio G., Thissera B., Orfali R., Jiffri S.E., Yaseen M., Sayed A.M., Rateb M.E. (2022). Neoechinulin A as a Promising SARS-CoV-2 Mpro Inhibitor: In Vitro and in Silico Study Showing the Ability of Simulations in Discerning Active from Inactive Enzyme Inhibitors. Mar. Drugs.

[54] Roby M.H.H., Abdelaliem Y.F., Esmail A.H.M., Mohdaly A.A.A., Ramadan M.F. (2020). Evaluation of Egyptian Honeys and Their Floral Origins: Phenolic Compounds, Antioxidant Activities, and Antimicrobial Characteristics. Environ. Sci. Pollut. Res..

[55] Hamdy A.A., Ismail H.M., Al-Ahwal A.E.-M.A., Gomaa N.F. (2009). Determination of Flavonoid and Phenolic Acid Contents of Clover, Cotton and Citrus Floral Honeys. J. Egypt. Public Health Assoc..

[56] Roby M.H.H., Sarhan M.A., Selim K.A.H., Khalel K.I. (2013). Evaluation of Antioxidant Activity, Total Phenols and Phenolic Compounds in Thyme (*Thymus vulgaris* L.), Sage (*Salvia officinalis* L.), and Marjoram (*Origanum majorana* L.) Extracts. Ind. Crops Prod..

[57] Ahmad Khera R., Nadeem F., Idrees Jilani M. (2016). Essential Chemical Constituents and Medicinal Uses of Marjoram (*Origanum majorana* L.)—A Comprehensive Review. Ijcbs.

[58] Abou-Shaara H.F. (2015). Potential Honey Bee Plants of Egypt. Cercet. Agron. Mold..

[59] Castro-Vázquez L., Díaz-Maroto M.C., González-Viñas M.A., Pérez-Coello M.S. (2009). Differentiation of Monofloral Citrus, Rosemary, Eucalyptus, Lavender, Thyme and Heather Honeys Based on Volatile Composition and Sensory Descriptive Analysis. Food Chem..

[60] Castro-Vázquez L., Díaz-Maroto M.C., Pérez-Coello M.S. (2007). Aroma Composition and New Chemical Markers of Spanish Citrus Honeys. Food Chem..

[61] Alissandrakis E., Daferera D., Tarantilis P.A., Polissiou M., Harizanis P.C. (2003). Ultrasound-Assisted Extraction of Volatile Compounds from Citrus Flowers and Citrus Honey. Food Chem..

[62] Jeong C.H., Jeong H.R., Choi G.N., Kim D.O., Lee U., Heo H.J. (2011). Neuroprotective and Anti-Oxidant Effects of Caffeic Acid Isolated from Erigeron Annuus Leaf. Chin. Med..

[63] Lim E.-K., Higgins G.S., Li Y., Bowles D.J. (2003). Regioselectivity of Glucosylation of Caffeic Acid by a UDP-Glucose: Glucosyltransferase Is Maintained in Planta. Biochem. J..

[64] Sambandam B., Thiyagarajan D., Ayyaswamy A., Raman P. (2016). Extraction and Isolation of Flavonoid Quercetin from the Leaves of Trigonella Foenum-Graecum and Their Anti-Oxidant Activity. Int. J. Pharm. Pharm. Sci..

[65] Selvaraj K., Chowdhury R., Bhattacharjee C. (2013). Isolation and Structural Elucidation of Flavonoids from Aquatic Fern Azolla Microphylla and Evaluation of Free Radical Scavenging Activity. Int. J Pharm. Pharm. Sci..

[66] Al-Ashaal H.A., El-Sheltawy S.T. (2011). Antioxidant Capacity of Hesperidin from Citrus Peel Using Electron Spin Resonance and Cytotoxic Activity against Human Carcinoma Cell Lines. Pharm. Biol..

[67] Choi J.S., Woo W.S., Young H.S., Park J.H. (1990). Phytochemical Study on Prunus Davidiana. Arch. Pharmacal Res..

[68] Gilbert N.C., Gerstmeier J., Schexnaydre E.E., Börner F., Garscha U., Neau D.B., Werz O., Newcomer M.E. (2020). Structural and Mechanistic Insights into 5-Lipoxygenase Inhibition by Natural Products. Nat. Chem. Biol..

[69] Kim S., Oshima H., Zhang H., Kern N.R., Re S., Lee J., Roux B., Sugita Y., Jiang W., Im W. (2020). CHARMM-GUI Free Energy Calculator for Absolute and Relative Ligand Solvation and Binding Free Energy Simulations. J. Chem. Theory Comput..

[70] Russo A., Acquaviva R., Campisi A., Sorrenti V., Di Giacomo C., Virgata G., Barcellona M.L., Vanella A. (2000). Bioflavonoids as Antiradicals, Antioxidants and DNA Cleavage Protectors. Cell Biol. Toxicol..

[71] Pokorny J., Yanishlieva N., Gordon M.H. (2001). Antioxidants in Food: Practical Applications.

[72] Rajalakshmi D., Narasimhan S. (1996). Food Antioxidants: Technological, Toxicological, Health Perspective.

[73] Buratti S., Benedetti S., Cosio M.S. (2007). Evaluation of the Antioxidant Power of Honey, Propolis and Royal Jelly by Amperometric Flow Injection Analysis. Talanta.

[74] Blasa M., Candiracci M., Accorsi A., Piacentini M.P., Albertini M.C., Piatti E. (2006). Raw Millefiori Honey Is Packed Full of Antioxidants. Food Chem..

[75] Al M.L., Daniel D., Moise A., Bobis O., Laslo L., Bogdanov S. (2009). Physico-Chemical and Bioactive Properties of Different Floral Origin Honeys from Romania. Food Chem..

[76] Katalinic V., Milos M., Kulisic T., Jukic M. (2006). Screening of 70 Medicinal Plant Extracts for Antioxidant Capacity and Total Phenols. Food Chem..

[77] Zalibera M., Staško A., Šlebodová A., Jančovičová V., Čermáková T., Brezová V. (2008). Antioxidant and Radical-Scavenging Activities of Slovak Honeys–An Electron Paramagnetic Resonance Study. Food Chem..

[78] Viuda-Martos M., Ruiz-Navajas Y., Fernández-López J., Pérez-Álvarez J.A. (2008). Functional Properties of Honey, Propolis, and Royal Jelly. J. Food Sci..

[79] Osher E., Weisinger G., Limor R., Tordjman K., Stern N. (2006). The 5 Lipoxygenase System in the Vasculature: Emerging Role in Health and Disease. Mol. Cell. Endocrinol..

[80] Joshi Y.B., Praticò D. (2015). The 5-Lipoxygenase Pathway: Oxidative and Inflammatory Contributions to the Alzheimer’s Disease Phenotype. Front. Cell. Neurosci..

[81] Li C., Zhang W., Fang S., Lu Y., Zhang L., Qi L., Huang X., Huang X., Wei E. (2010). Baicalin Attenuates Oxygen-Glucose Deprivation-Induced Injury by Inhibiting Oxidative Stress-Mediated 5-Lipoxygenase Activation in PC12 Cells. Acta Pharmacol. Sin..

[82] Liu Y., Wang W., Li Y., Xiao Y., Cheng J., Jia J. (2015). The 5-Lipoxygenase Inhibitor Zileuton Confers Neuroprotection against Glutamate Oxidative Damage by Inhibiting Ferroptosis. Biol. Pharm. Bull..

[83] Maccarrone M., Meloni C., Manca-di-Villahermosa S., Cococcetta N., Casciani C.U., Finazzi-Agrò A., Taccone-Gallucci M. (2001). Vitamin E Suppresses 5-Lipoxygenase-Mediated Oxidative Stress in Peripheral Blood Mononuclear Cells of Hemodialysis Patients Regardless of Administration Route. Am. J. Kidney Dis..

[84] Mathew S., Abraham T.E. (2006). Studies on the Antioxidant Activities of Cinnamon (*Cinnamomum verum*) Bark Extracts, through Various in Vitro Models. Food Chem..

[85] Sancho M.T., Pascual-Maté A., Rodríguez-Morales E.G., Osés S.M., Escriche I., Periche Á., Fernández-Muiño M.A. (2016). Critical Assessment of Antioxidant-related Parameters of Honey. Int. J. Food Sci. Technol..

[86] Wu H.-C., Shiau C.-Y., Chen H.-M., Chiou T.-K. (2003). Antioxidant Activities of Carnosine, Anserine, Some Free Amino Acids and Their Combination. J. Food Drug Anal..

[87] Seeliger D., de Groot B.L. (2010). Ligand docking and binding site analysis with PyMOL and Autodock/Vina. J. Comput. Aided Mol. Des..

[88] Bowers K.J., Chow D.E., Xu H., Dror R.O., Eastwood M.P., Gregersen B.A., Klepeis J.L., Kolossvary I., Moraes M.A., Sacerdoti F.D. (2006). Scalable algorithms for molecular dynamics simulations on commodity clusters. Proceedings of the SC’06: Proceedings of the 2006 ACM/IEEE Conference on Supercomputing.

[89] Release S. (2017). 3: Desmond Molecular Dynamics System, DE Shaw Research, New York, NY, 2017.

[90] Phillips J.C., Braun R., Wang W., Gumbart J., Tajkhorshid E., Villa E., Chipot C., Skeel R.D., Kalé L., Schulten K. (2005). Scalable molecular dynamics with NAMD. J. Comput. Chem..

[91] Ngo S.T., Tam N.M., Quan P.M., Nguyen T.H. (2021). Benchmark of Popular Free Energy Approaches Revealing the Inhibitors Binding to SARS-CoV-2 Mpro. J. Chem. Inf. Model..

