# Supplementary material for: ¹H-NMR Metabolic Profiling, Antioxidant Activity, and Docking Study of Common Medicinal Plant-Derived Honey
Source: Antioxidants (Basel). 2022 Sep 22;11(10):1880. doi: 10.3390/antiox11101880 (PMC9598149; doi:10.3390/antiox11101880)
Supplement: Supplementary file 1 [file antioxidants-11-01880-s001.zip › antioxidants-1889956-supplementary.pdf]

Supplementary Material

[1]1H-NMR Metabolic Profiling, antioxidant activity and docking study of common medicinal plants-derived honey

Maha Montaser[1], Asmaa T. Ali[2], Ahmed M. Sayed[1], Usama Ramadan Abdelmohsen[3,4], Ehab W. Zidan[5], Raha Orfali[6], Mostafa E. Rateb[7], Mohamed A. Zaki[8], Hossam M. Hassan[8*], Rabab Mohammed[8*] and Mohamed S. Hifnawy[9].

[1] Department of Pharmacognosy, Faculty of Pharmacy, Nahda University, Beni-Suef 62513, Egypt
[2] Department of Biochemistry, Faculty of Pharmacy, Nahda University, Beni-Suef 62511, Egypt
[3] Department of Pharmacognosy, Faculty of Pharmacy, Minia University, Minia 61519, Egypt
[4] Department of Pharmacognosy, Faculty of Pharmacy, Deraya University, New Minia City 61111, Egypt
[5] Department of Bee Research, Plant Protection Research Institute, Agricultural Research Centre,
Giza 12618, Egypt
[6] Department of Pharmacognosy, College of Pharmacy, King Saud University, Riyadh 11451, Saudi Arabia
[7] School of Computing, Engineering & Physical Sciences, University of the West of Scotland, Paisley PA1 2BE, UK
[8] Department of Pharmacognosy, Faculty of Pharmacy, Beni-Suef University, Beni-Suef 62514, Egypt
[9] Department of Pharmacognosy, Faculty of Pharmacy, Cairo University, Cairo 11787, Egypt
* Correspondence: hossam.mokhtar@nub.edu.eg (H.M.H.); rababmohammed@pharm.bsu.edu.eg (R.M.)

1.1 Molecular Docking

AutoDock Vina software was used in all molecular docking experiments [1]. All isolated compounds were docked against the 5-LOX crystal structure (PDB codes: 6N2W). The binding site was determined according to the enzyme's co-crystallized ligand (NDGA). The co-ordinates of the grid box were: x = -12.87; y = 16.3; z = 68.64. The size of the grid box was set to be 10 Å. Exhaustiveness was set to be 24. Ten poses were generated for each docking experiment. Docking poses were analysed and visualized using Pymol software [1].

1.2 Molecular Dynamics Simulation

Desmond v. 2.2 software was used for performing MDS experiments [2–4]. This software applies the OPLS force field. Protein systems were built using the System Builder option, where the protein structure was embedded in an orthorhombic box of TIP3P water together with 0.15 M Na^+ and Cl^- ions in 20 Å solvent buffer. Afterward, the prepared systems were energy minimized and equilibrated for 10 ns. Desmond software automatically parameterizes inputted ligands during the system building step according to the OPLS force field. Metal-containing proteins like 5-LOX that contain histidine-Fe^{+2} complex in the active site should be parameterized during the protein preparation step. To do so, a hetero state should be generated for hetero atoms like Fe (Generate Hetero States). This function is a part of the maestro's Protein Preparation wizard. This step will enable the formation of a suitable hetero state or co-ordinate covalent state for the heteroatom (i.e. Fe^{+2}) in complex with the protein so that force fields like OPLS can easily recognize the zinc atom. For simulations performed by NAMD [5], the parameters and topologies of the compounds were calculated either using the Charmm27 force field with the online software Ligand Reader and Modeler (http://www.charmm-gui. org/?doc=input/ligandrm) [6] or using the VMD plugin Force Field Toolkit (ffTK). Afterward, the generated parameters and topology files were loaded to VMD to readily read the protein–ligand complexes without errors and then conduct the simulation step. Harmonic Tcl forces were applied to keep Fe^{+2} in place.

1.3 Binding Free Energy Calculations

Binding free energy calculations (ΔG) were performed using the free energy perturbation (FEP) method [5]. This method was described in detail in the recent article by Kim and coworkers [5]. Briefly, this method calculates the binding free energy ΔGbinding according to the following equation: ΔGbinding = ΔGComplex - ΔGLigand. The value of each ΔG is estimated from a

separate simulation using NAMD software. Interestingly, all input files required for simulation by NAMD can be prepared by using the online website CharmmGUI (https://charmm gui.org/?doc=input/afes.abinding). Subsequently, we can use these files in NAMD to produce the required simulations using the FEP calculation function in NAMD. The equilibration was achieved in the NPT ensemble at 300 K and 1 atm (1.01325 bar) with Langevin piston pressure (for "Complex" and "Ligand") in the presence of the TIP3P water model. Then, 10 ns FEP simulations were performed for each compound, and the last 5 ns of the free energy values was measured for the final free energy values [4]. Finally, the generated trajectories were visualized and analyzed using VMD software. It worth noting that Ngo and co-workers in their recent benchmarking study found that the FEP method of determination of ΔG was the most accurate method in terms of predicting enzyme inhibitors [6].

2- ^{1}H-NMR analysis

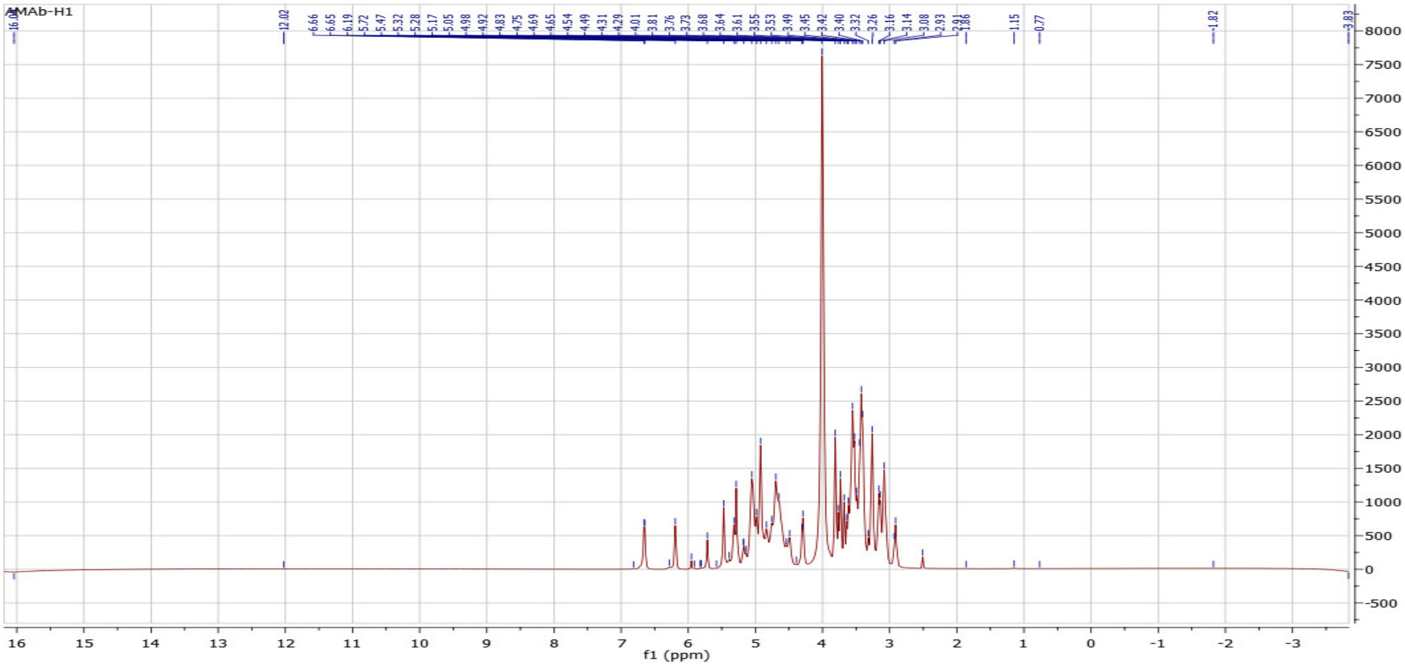

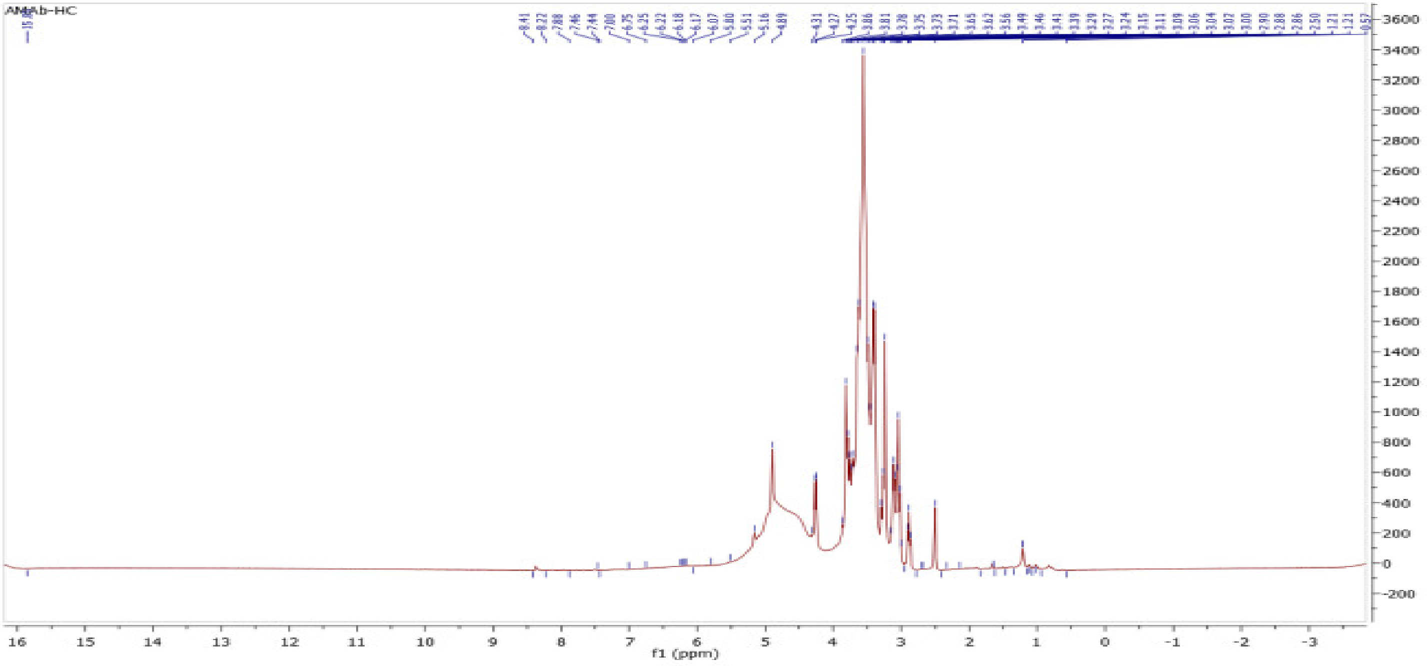

Figure S1. ^{1}HNMR chart of Citrus honey extract and its 2nd metabolites (H1and Hc).

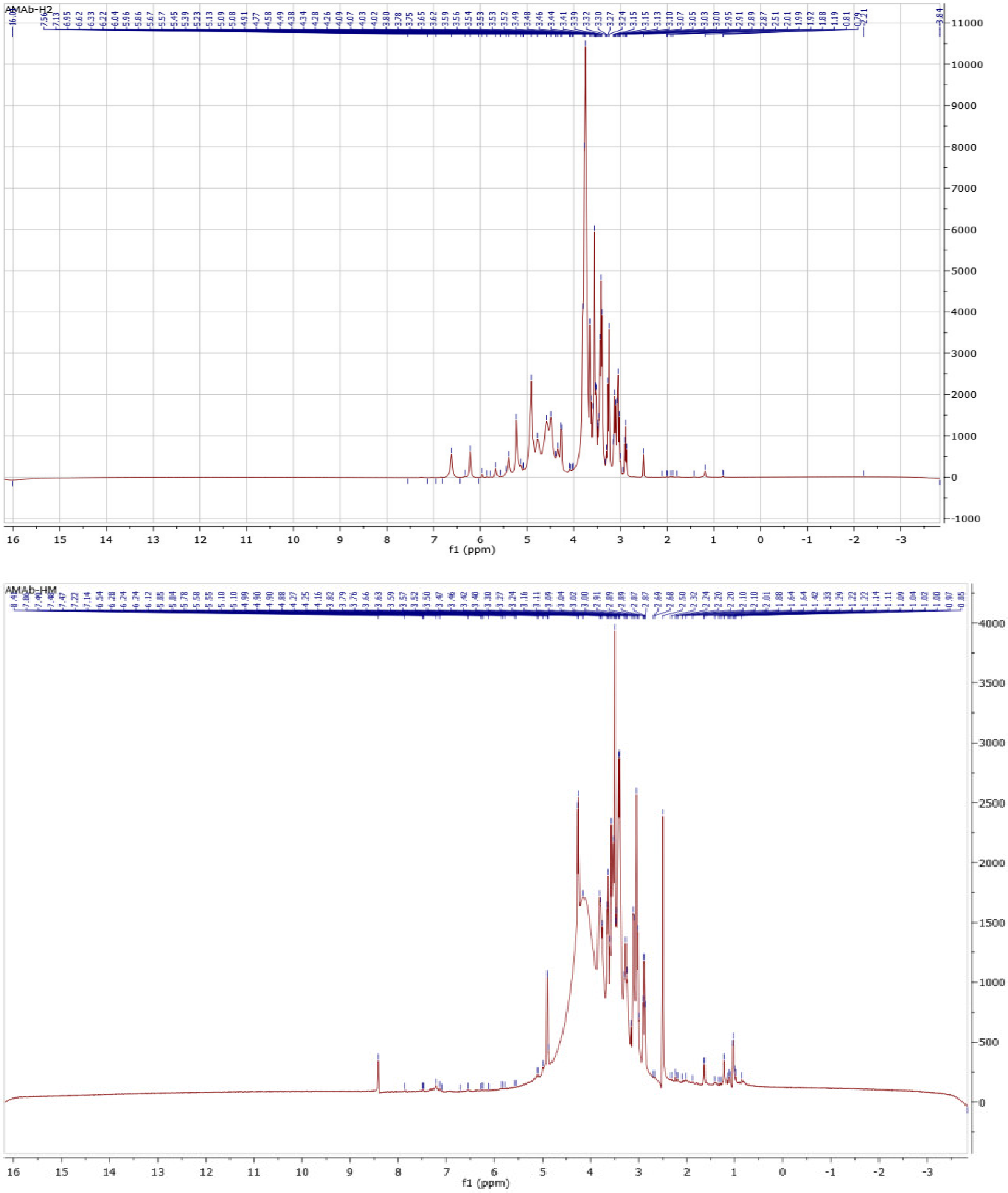

Figure S2. ^{1}HNMR chart of Marjoram honey extract and its 2nd metabolites (H2and HM).

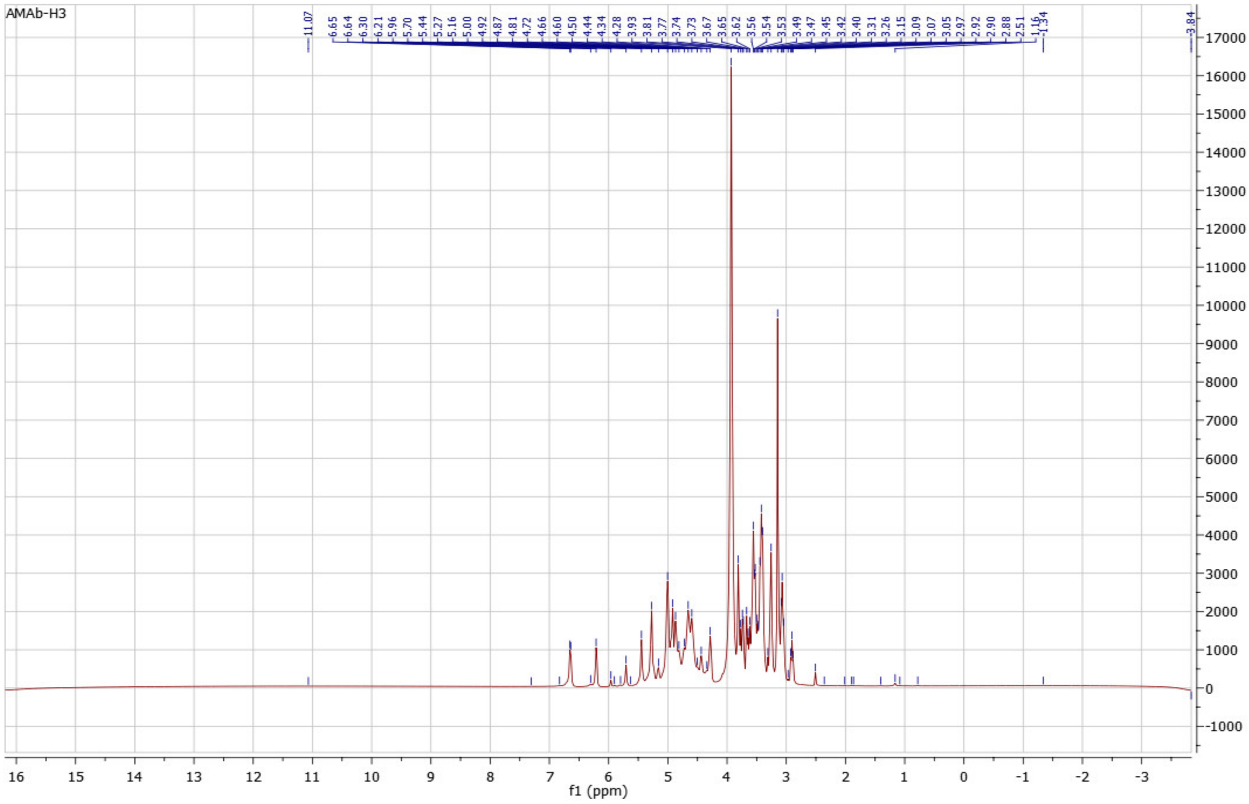

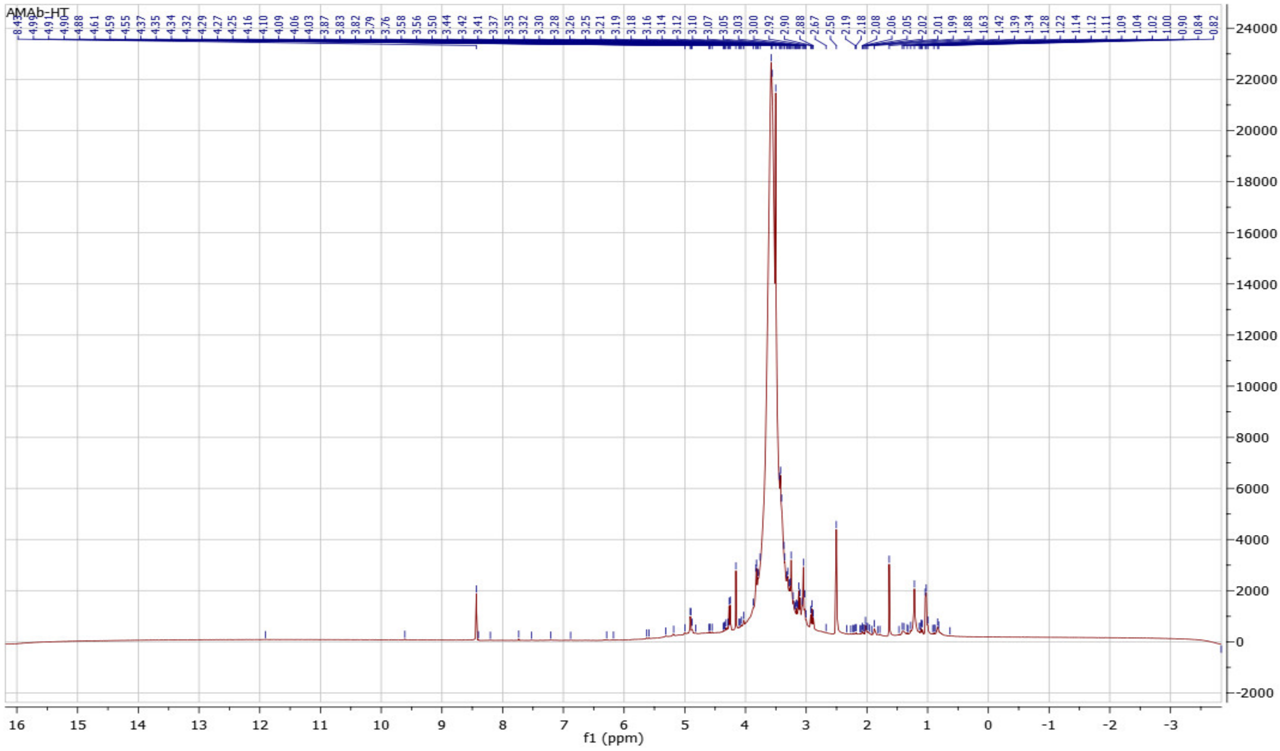

Figure S3. ^{1}HNMR chart of Clover honey extract and its 2nd metabolites (H3and HT).

3- Identification of isolated compounds

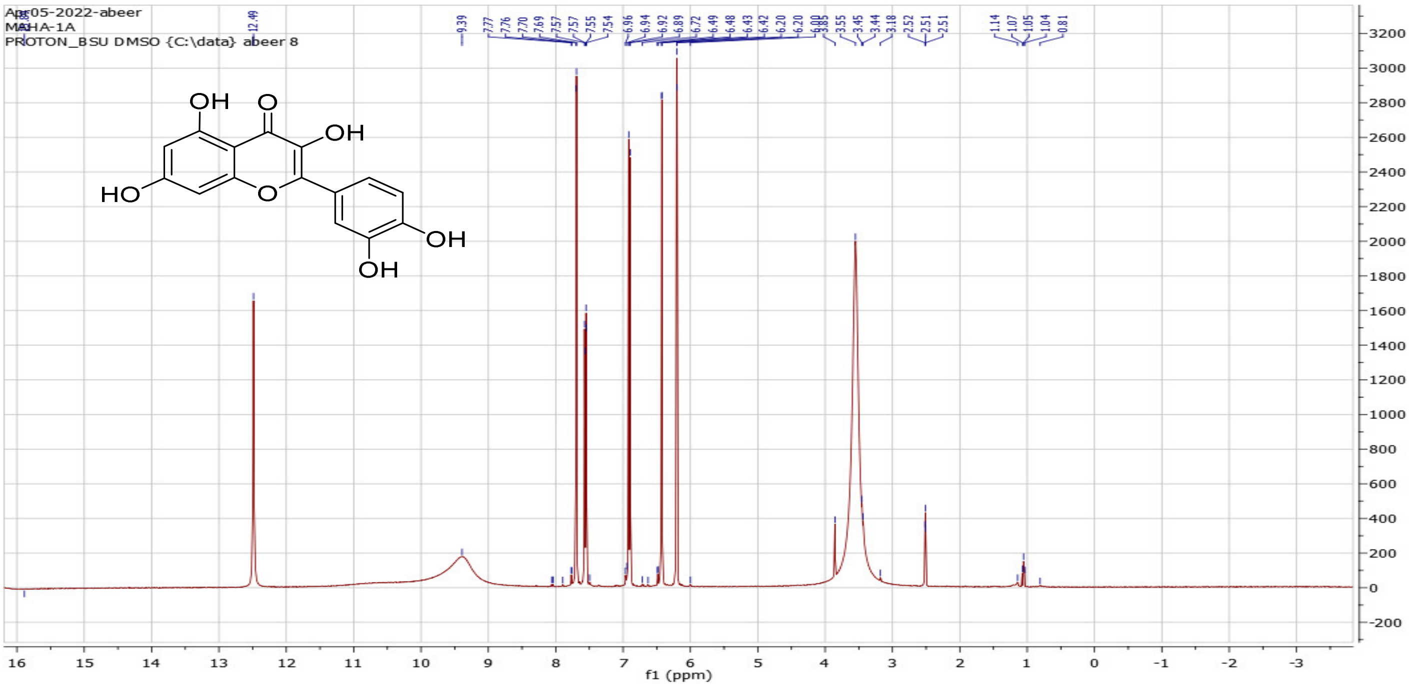

Figure S4. ¹HNMR chart of compound no 2 : Qurcetin.

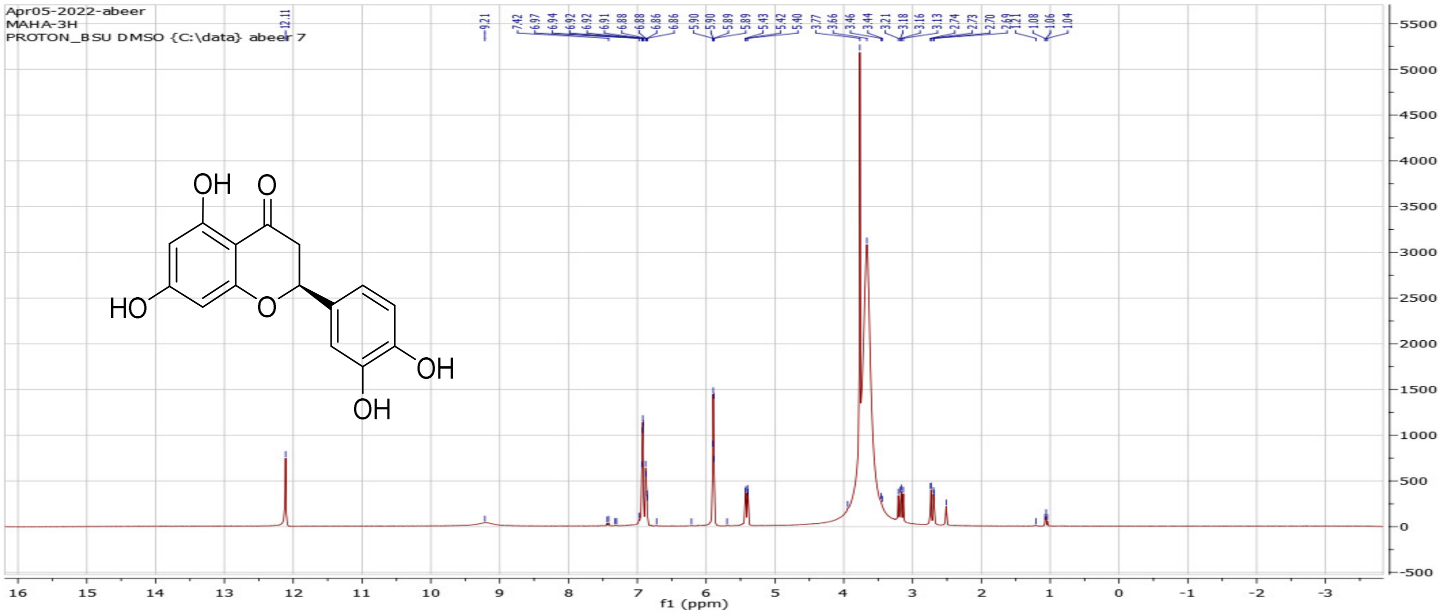

Figure S5. ¹HNMR chart of compound no 3 : Hesperetin.

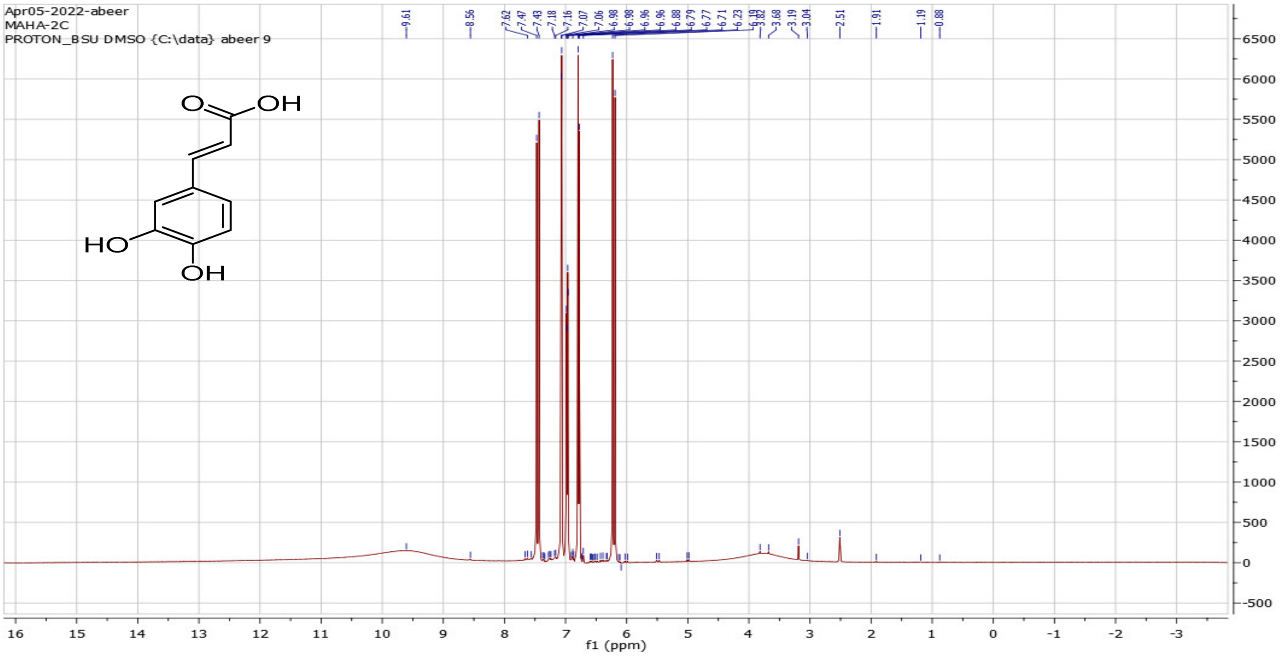

Figure S6. ¹HNMR chart of compound no 1 : Caffeic acid.

References

1. Seeliger, D.; de Groot, B.L. Ligand docking and binding site analysis with PyMOL and Autodock/Vina. J. Comput. Aided Mol. Des. 2010, 24, 417–422.

2. Bowers, K.J.; Chow, D.E.; Xu, H.; Dror, R.O.; Eastwood, M.P.; Gregersen, B.A.; Klepeis, J.L.; Kolossvary, I.; Moraes, M.A.; Sacerdoti, F.D.; et al. Scalable algorithms for molecular dynamics simulations on commodity clusters. In Proceedings of the SC'06: Proceedings of the 2006 ACM/IEEE Conference on Supercomputing, Tampa, FL, USA, 11–17 November 2006; IEEE: New York, NY, USA, 2006; p. 43.

3. Release, S. 3: Desmond Molecular Dynamics System, DE Shaw Research, New York, NY, 2017; Maestro-Desmond Interoperability Tools, Schrödinger: New York, NY, USA, 2017.

7. Schrodinger LLC. Maestro, Version 9.0; Schrodinger LLC: New York, NY, USA, 2009.

4. Phillips, J.C.; Braun, R.; Wang, W.; Gumbart, J.; Tajkhorshid, E.; Villa, E.; Chipot, C.; Skeel, R.D.; Kalé, L.; Schulten, K. Scalable molecular dynamics with NAMD. J. Comput. Chem. 2005, 26, 1781–1802.

5. Kim, S.; Oshima, H.; Zhang, H.; Kern, N.R.; Re, S.; Lee, J.; Rous, B.; Sugita, Y.; Jiang, W.; Im, W. CHARMM-GUI free energy calculator for absolute and relative ligand solvation and binding free energy simulations. J. Chem. Theory Comput. 2020, 16, 7207–7218.

6. Ngo, S.T.; Tam, N.M.; Quan, P.M.; Nguyen, T.H. Benchmark of Popular Free Energy Approaches Revealing the Inhibitors Binding to SARS-CoV-2 Mpro. J. Chem. Inf. Model. 2021, 61, 2302–2312.
